# Unintended Pregnancy and Its Correlates among Female Attendees of Sexually Transmitted Disease Clinics in Eastern China

**DOI:** 10.1155/2013/349174

**Published:** 2013-06-13

**Authors:** Qiaoqin Ma, Xiaohong Pan, Gaofeng Cai, Jiezhe Yan, Yun Xu, Masako Ono-Kihara, Masahiro Kihara

**Affiliations:** ^1^Department of HIV/STD Control, Zhejiang Provincial Center for Disease Prevention and Control, Hangzhou 310051, China; ^2^Department of Global Health and Socio-Epidemiology, Kyoto University School of Public Health, Kyoto 606-8501, Japan

## Abstract

This study is to determine the prevalence of unintended pregnancy and its risk factors among the female attendees of sexually transmitted disease (STD) clinics in Zhejiang Province, China. A self-administered questionnaire survey of a cross-sectional design was administered to attendees at four STD clinics in 2007. Of the 313 female STD clinic attendees, 42.5% reported that they had at least one unintended pregnancy; the induced abortion rate was 39.0%. Over their lifetime, 12.1% responded “use condoms always/often” and 5.4% “always/often used oral contraceptives.” The risk factors for the unintended pregnancy identified by the multivariate analysis were as follows: being married, experience of nonconsensual sex, and a history of STD, having two and over two sexual partners. Unintended pregnancies and induced abortion by female STD clinic attendees have reached an alarming prevalence. Doctors at STD clinics should attach importance not only to the STD problem of the female attendees, but also to the unintended pregnancy and the associated factors. Targeted contraceptive counseling and intervention should be promoted at STD clinics as a strategy to improve the efficiency and effectiveness of the reproductive health services in China.

## 1. Introduction

Unintended pregnancy is one of the principal reproductive health problems in China; many Chinese females have unintended pregnancies and suffer the consequences. A recent study revealed that 9.1% of the Chinese married females of childbearing age had an unintended pregnancy during the previous year [1]. According to a Health Ministry report, 6–10 million Chinese females underwent induced abortions annually between 2000 and 2009 [2]. In China, reproductive health care is easily accessed through family planning programs that provide contraceptive counseling and education, various contraception services, and induced abortion when necessary. Oral contraceptives (OCs) and condoms are widely available at drug stores and supermarkets. Information on the risk factors for unintended pregnancy is critical for improving the reproductive health services, to address th0e gap between the wide availability of contraception and the high prevalence of unintended pregnancy and its consequences. However, little research has examined this in China; the few studies extant focused on university students [3, 4], and females of childbearing age [1] or seeking abortions [5].

The incidences of sexually transmitted diseases (STDs) have increased rapidly in China. The national reported incidence of syphilis increased from 0.09 per 100,000 in 1990 to 23.07 per 100,000 in 2009 [6]. Of the 28 notifiable infectious diseases in China, syphilis ranks the third and gonorrhea the sixth in terms of both incident cases and incidence rate [2]. With the increase in STD transmission in China, STDs might be more likely to occur in conjunction with unintended pregnancy, as both are due to the consequences of unprotected sex. A high prevalence of STDs has been identified among pregnant females at antenatal, abortion, and family planning clinics in both China [7–9] and elsewhere [10–12].

Chinese studies have documented that STD clinic attendees rarely protect themselves with condoms during their sexual intercourse, and many are infected with STDs [13, 14]; however, there are no reports of the prevalence of contraception or unintended pregnancy in this population. Therefore, we examined the risks of unintended pregnancy among female attendees at four STD clinics in Zhejiang Province, where the reported incidences of syphilis and gonorrhea are among the highest in China [15].

## 2. Methods

### 2.1. Participants and Data Collection

The research method was introduced somewhere else [16]. The participants of this study came from a cross-sectional survey conducted at four STD clinics in Zhejiang Province, Eastern China, from October to December, 2007. In 2007, 12 human immunodeficiency virus (HIV) surveillance sentinels were established at STD clinics in Zhejiang Province, to survey the prevalence of HIV and core information on their behaviors related to HIV transmission in the 3 months from April to June. Of the 12, four STD clinics agreed to conduct this study after reviewing the research protocol. We compared the attendees' gender, age, marital status, and residence between the four participating STD clinics and the remaining eight clinics using the 2007 surveillance data; the demographic characteristics were similar in the two groups. In principle, the study enrolled all sexually active attendees visiting the STD clinics for the diagnosis and treatment of STD, older than 14 years. Those attendees who were not sexually active, unwilling to participate in the study, had a language barrier, or visited the clinics for general skin diseases were excluded.

This paper only includes female STD clinic attendees. In total, 466 females visited the clinics for STD problems during the study period, and 334 females agreed to participate in the study with a response rate of 71.7%. Of the 334 females, 322 gave valid responses. Of these, 6 females only ever had sex with the same sex partner. We omitted these 6 individuals from further analyses because their sexual intercourse evidently could not lead to pregnancy, resulting in a sample size of 316. Those attendees who completed the question on their history of unintended pregnancy over a lifetime were included in the analysis, giving a final sample size of 313 females.

The questionnaire used in this study was developed after a review of the domestic and international literature, and it was modified after repeated discussion among the research team and doctors/nurses at the clinics studied. The final questionnaire had five sections with 7, 10, 21, 8, and 5 questions, respectively. The questionnaires were self-administered and anonymous, and they were collected consecutively by doctors or nurses at the clinics from October to December 2007.

### 2.2. Ethical Considerations

All attendees of the four clinics who met the recruitment criteria were informed of the study purpose and method and that participant privacy and confidentiality would be strictly protected. This information was also printed at the beginning of the questionnaire. This research was ratified by the Zhejiang Provincial Health Ministry. Those responsible for institutional review at Zhejiang Province's Center for Disease Control and Prevention and the four STD clinics approved this research and the study protocol.

### 2.3. Statistical Analysis

The participants were divided into two groups: those who had experienced unintended pregnancy and those who had not.

Participants' experience of unintended pregnancy was used as the dependent variable in the analysis. The independent variables included sociodemographic parameters, variables related to sexual behavior during (i) the first sexual activity, (ii) the participant's lifetime and current time, and (iii) the previous 6 months.

Factors associated with unintended pregnancy were identified using univariate and multivariate logistic regression analyses. Variables significant (*P* value <0.05) in the univariate analyses, other than those for the previous 6 months, were included in the multivariate models with participant's age, income, education level, marital status, and current employment being fixed in the models. Multivariate analyses were performed using a backward stepwise logistic regression analysis with a *P* value >0.10 as the removal criterion. A *P* value <0.05 was regarded as statistically significant. The data were analyzed using SPSS for Windows (ver. 17.0; SPSS, Chicago, IL, USA).

## 3. Results

Of the 313 female attendees, 133 (42.5%) reported a history of unintended pregnancy and 122 (39.0%) reported a history of induced abortion. The mean ± standard deviation (SD) number of unintended pregnancies was 1.52 ± 0.86 (range: 1–6); the mean number of induced abortions was 1.49 ± 0.87 (range: 1–6).

Of the participants' variables, age was distributed roughly evenly (Table 1). A percentage of 66.1% of the participants earned less than 2000 renminbi (RMB) per month, 72.8% had not finished high school at most, and 66.5% were married. Regarding the employment status, 29% of the participants were unemployed, 7% were retired, and 8.9% worked in the sector of public service. According to the univariate analysis, participant's age, education level, and marital status were unrelated to unintended pregnancy. Earning a salary ≥ 2000 RMB and having worked in the public sector were associated with unintended pregnancy.

Various sexual behaviors at first sex, lifetime, the previous 6 months, and diagnosed STD at lifetime and current time are shown in Table 2. The rate of always/often condom use over the lifetime was 12.1%, while the percentage for OC use was 5.4%. Multiple sexual partners were prevalent, and only 53.4% of participants reported only one partner during their lifetimes.

With respect to risks associated with unintended pregnancy (Table 2), female STD clinic participants who initiated sex at a younger age (versus those who initiated later), had nonconsensual sex (versus those with no such history), reported having had been diagnosed with an STD (versus those with no such history), having had two or more sexual partners (versus those having only one), rarely/sometimes and always/often used condoms (versus those who never used them), rarely/sometimes used OC (versus those who never used OC), were more likely to report a history of unintended pregnancy during their lifetime. Participants who had more than two sexual partners (versus those having only one), participants who rarely/sometimes and always/often used condoms (versus those who never used them), and participants who rarely/sometimes used OC (versus those who never used them) during the previous 6 months were associated with unintended pregnancy. Condom use and OC use at first sex, current STD diagnosed, and type of sex during lifetime were not associated with unintended pregnancy.

Multivariate logistic regression analysis revealed that being married (odds ratio (OR) 2.99, 95% confidence interval (95% CI) 1.12–7.96), experience of nonconsensual sex (OR 5.83, 95% CI 1.74–19.69), a history of STD (OR 3.31, 95% CI 1.82–6.01), and having two (OR 2.09, 95% CI 1.08–4.05) or more sexual partners (OR 3.02, 95% CI 1.44–6.35) remained risk factors for a history of unintended pregnancy (Table 3). However, the association of age at first sex, condom use, and OC use with unintended pregnancy disappeared in the multivariate analysis. 

## 4. Discussion

This study addressed the gap in knowledge regarding unintended pregnancy and examined the associated factors among women attending four STD clinics. To our knowledge, this is the first quantitative study of its kind conducted in China. We found that the rate of unintended pregnancy among female STD clinic attendees was 42.5%. The amazingly high rate of unintended pregnancy in this study implies that our participants make poor use of not only barrier methods (such as condoms) but also other effective contraception, putting them at risk of both STDs and unintended pregnancy.

Research has indicated that using condoms correctly and consistently reduces substantially the consequences of unprotected intercourse [17, 18]. We identified a very low rate of condom use; only 12% always or often used condoms during their lifetimes, and around 9% in the previous 6 months. The proportion of our subjects using condom is clearly too small to prevent STDs and pregnancy. Surprisingly, those who always/often or rarely/sometimes used condoms during their lifetimes were more likely to experience unintended pregnancy compared with those who never used condoms in the bivariate analysis; though this correlation did not remain after adjustment for possible confounding, it seems that condom use does not reduce the risk of unintended pregnancy in this study. Those who never used condoms may have adopted other effective contraception, such as an intrauterine device (IUD) or sterilization; consequently, their pregnancy rate was not higher than those who used condoms; furthermore, many international and domestic studies have revealed that condom use was not effective enough in terms of preventing pregnancy, and they, in addition, highlighted the importance of correct condom use every time partners have sexual intercourse [19–21].

The consequences of low condom use are not limited to unintended pregnancy, but they naturally include an increased risk of STDs. A percentage of 39% of the participants was diagnosed with a current STD. The reported history of diagnosed STD reached 28%. We found that those who had a history of STD were more likely to report a history of unintended pregnancy, suggesting that female STD clinic attendees use little contraception other than condom use. 

Male condoms are the leading contraceptive method for unmarried young Chinese, followed, in order, by withdrawal, rhythm methods, and OC [22–24]. The rate of contraception use is high for married people, who mainly use IUD, sterilization, condom, or OC [25]. However, these findings do not apply to our study. Of our subjects, around 5% reported using OC always or often during their lifetimes and in the previous 6 months, which is even a much lower rate than that of condom use. High prevalence of unintended pregnancy and STD in our participants implies that the majority of our participants use not only condom and OC but also any other contraception either never or rarely. Chinese studies revealed that 50%–60% of females who become pregnant unintentionally and seek an induced abortion at a hospital did not use contraception [26, 27]. Doctors at STD clinics therefore should relay the importance of contraception to STD clinic attendees, specifically to STD-infected attendees, because they are at elevated risk of unintended pregnancy. 

The rate and mean number of induced abortions in this study were slightly lower than that of unintended pregnancy. We speculate that the majority of unintended pregnancies were aborted artificially. The reason our pregnant participants chose abortion was unclear. This might be because some pregnancies occur with nonstable sexual partners. We found a high prevalence of multiple sexual partners among the participants during their lifetimes and even during the previous 6 months. The rate of abortion might also be related to the one-child policy among those who already had one child [28]. The high rates of unintended pregnancy and induced abortion emphasize the need for doctors at STD clinics to play an expanded role in sexual behavior intervention related to STDs to minimize these risks. The receipt of contraceptive counseling was associated with greater self-efficacy and increased contraception use to prevent unintended pregnancy [29, 30]. Therefore, contraception counseling should be considered a component of behavioral intervention at STD clinics.

We also found that having multiple sexual partners was a risk factor for unintended pregnancy, consistent with the results in Chinese university students [3]. The trend that the more sexual partners our female participants had the more likely they experienced an unintended pregnancy indicates that an increased number of sexual partners of those female participants did not result in increased contraceptive use. The fact that multiple sexual partnerships are very prevalent heightens the concern of unintended pregnancy for this population. 

A history of nonconsensual sex was strongly related to unintended pregnancy in female attendees of STD clinics; this had the highest odds ratios for our participants, corroborating reports in other domestic and foreign populations [3, 31, 32]. Nonconsensual sex has also been reported to be associated with other risks, such as more sex partners, reduced condom use, and an increased risk of contracting STD/HIV [33–35]. These findings indicate that a history of nonconsensual sex in attendees at STD clinics warrants special consideration, and those so identified should be referred for appropriate counseling and reproductive health services.

Our study had several limitations. Its cross-sectional nature precluded determination of causal associations with unintended pregnancy; thus, further studies to identify factors associated with unintended pregnancy are necessary to better understand the associations we report here. Bias might have been introduced by the refusal of some attendees to participate. Self-reported sexual behaviors and STD histories might have been under-reported due to their sensitive nature in the Chinese culture, but this might not be evident because requesting an STD diagnosis and treatment at an STD clinic implies the acknowledgement of risky sexual behaviors. Finally, findings from four STD clinics might not be representative of STD clinic attendees in either this one province or all of China. However, we compared the demographic data of our study population and those females from the sentinel surveillance at 12 STD clinics in 2007, and we found that the distributions of age, marital status, and residence were all similar.

## 5. Conclusion

This study provides valuable information on unintended pregnancy in STD clinics in China. Female attendees seen in STD clinics feebly protect themselves during sexual intercourse, and they are vulnerable to both STDs and unintended pregnancy. The factors leading our attendees to expose themselves to these dual risks should be considered as an integral part of the development of prevention strategies. Doctors at STD clinics should attach importance to not only the STD problem of the attendees but also the unintended pregnancy and the associated factors. Contraceptive counseling and intervention should be promoted at STD clinics as a strategy to improve the efficiency and effectiveness of reproductive health services in China.

## Figures and Tables

**Table 1:** Demographic characteristics for unintended pregnancy among female STD clinic attendees.

Variable	Total (%)^a^	Pregnancy (%)	Crude OR (95% CI)^b^	*P* value
Age				
<30	169 (54.0)	70 (41.4)	1	
≥30	144 (46.0)	63 (43.8)	1.10 (0.70–1.72)	0.678
Income per month				
<2000	207 (66.1)	85 (41.1)	1	
≥2000	50 (16.0)	31 (62.0)	2.34 (1.24–4.42)	0.009
Education				
Below high school	228 (72.8)	103 (45.2)	1	
High school and above	84 (26.8)	29 (34.5)	0.64 (0.38–1.08)	0.092
Marriage				
Single	44 (14.1)	15 (34.1)	1	
Cohabitation	55 (17.6)	25 (45.5)	1.61 (0.71–3.65)	0.254
Married	208 (66.5)	90 (43.3)	1.48 (0.75–2.91)	0.264
Employment status				
Unemployed	90 (28.8)	29 (32.2)	1	
Public	28 (8.9)	18 (64.3)	3.79 (1.55–9.23)	0.003
Retired	23 (7.3)	12 (52.2)	2.30 (0.91–5.82)	0.080
Others	169 (54.0)	73 (43.2)	1.60 (0.94–2.74)	0.086

^a^Percentages may not add up to 100 due to missing data; ^b^OR: odds ratio; CI: confidence interval.

**Table 2:** Bivariate correlates of unintended pregnancy with sexual behaviors.

Variable	Total (%)^a^	Pregnancy (%)	Crude OR (95% CI)^b^	*P* value
Age of first sex				
≥20	190 (60.7)	70 (36.8)	1	
<20	117 (37.4)	61 (52.1)	1.87 (1.17–2.98)	0.009
Condom use for first sex				
Use	28 (8.9)	9 (32.1)	1	
Nonuse/forget	281 (89.8)	122 (43.4)	1.62 (0.71–3.71)	0.253
OC use first sex				
Use	24 (7.7)	11 (45.8)	1	
Nonuse/forget	285 (91.1)	120 (42.1)	0.86 (0.37–1.98)	0.723
Ever nonconsent sex				
No	282 (90.1)	107 (37.9)	1	
Yes	29 (9.3)	24 (82.8)	7.85 (2.91–21.19)	0.000
STD history				
No	218 (69.6)	74 (33.9)	1	
Yes	87 (27.8)	54 (62.1)	3.18 (1.90–5.33)	0.000
Current STD				
No	189 (60.4)	80 (42.3)	1	0.958
Yes	122 (39.0)	52 (42.6)	1.01 (0.64–1.60)	
Partner number over lifetime				
1	166 (53.4)	54 (32.5)	1	
2	67 (21.5)	31 (46.3)	1.79 (1.00–3.19)	0.050
≥3	69 (22.2)	44 (63.8)	3.65 (2.03–6.58)	0.000
Type of sex over lifetime				
Only vaginal	293 (93.6)	122 (41.6)	1	
Ever anal or oral	13 (4.2)	8 (61.5)	2.24 (0.72–7.02)	0.165
Condom use over lifetime				
Never	99 (31.6)	26 (26.3)	1	
Rarely/sometimes	171 (54.6)	86 (50.3)	2.84 (1.66–4.87)	0.000
Always/often	38 (12.1)	20 (52.6)	3.12 (1.43–6.79)	0.004
OC use over lifetime				
Never	144 (46.0)	51 (35.4)	1	
Rarely/sometimes	145 (46.3)	74 (51.0)	1.90 (1.19–3.05)	0.008
Always/often	17 (5.4)	7 (41.2)	1.28 (0.46–3.56)	0.641
Partner number half year^c^				
1	178 (76.1)	74 (41.6)	1	
2	30 (12.8)	11 (36.7)	0.81 (0.37–1.81)	0.613
≥3	18 (7.7)	13 (72.2)	3.65 (1.25–10.69)	0.018
Condom use half year^c^				
Never	101 (43.0)	32 (31.7)	1	
Rarely/sometimes	110 (46.8)	56 (50.9)	2.24 (1.28–3.92)	0.005
Always/often	22 (9.4)	12 (54.5)	2.59 (1.01–6.61)	0.047
OC use half year^c^				
Never	144 (61.3)	54 (37.5)	1	
Rarely/sometimes	74 (31.5)	38 (51.4)	1.76 (1.00–3.10)	0.050
Always/often	11 (4.7)	7 (63.6)	2.92 (0.82–10.43)	0.100

^a^Percentages may not add up to 100 due to missing data; ^b^OR: odds ratio; CI: confidence interval.

^c^235 of females were sexually active in the previous half year.

**Table 3:** Multivariate analysis predicting unintended pregnancy.

Variable	Adjusted OR (95% CI)^a^	*P* value
Marriage		
Single	1	
Cohabitation	2.55 (0.93–6.96)	0.068
Married	2.99 (1.12–7.96)	0.028
Ever nonconsent sex		
No	1	
Yes	5.83 (1.74–19.69)	0.004
STD history		
No	1	
Yes	3.31 (1.82–6.01)	0.000
Partner number over lifetime		
1	1	
2	2.09 (1.08–4.05)	0.029
≥3	3.02 (1.44–6.35)	0.004

^a^OR: odds ratio; CI: confidence interval.
